# Comparison of heat-sensitive moxibustion versus fluticasone/salmeterol (seretide) combination in the treatment of chronic persistent asthma: design of a multicenter randomized controlled trial

**DOI:** 10.1186/1745-6215-11-121

**Published:** 2010-12-15

**Authors:** Rixin Chen, Mingren Chen, Jun Xiong, Fan Yi, Zhenhai Chi, Bo Zhang

**Affiliations:** 1 The Affiliated Hospital of Jiangxi University of TCM, Nanchang, PR China; 2 Department of Health of Jiangxi Province, Nanchang, PR China

## Abstract

**Background:**

Asthma is a major health problem and has significant mortality around the world. Although the symptoms can be controlled by drug treatment in most patients, effective low-risk, non-drug strategies could constitute a significant advance in asthma management. An increasing number of patients with asthma are attracted by acupuncture and moxibustion. Therefore, it is of importance that scientific evidence about the efficacy of this type of therapy is regarded. Our past researches suggested heat-sensitive moxibustion might be effective in treatment of asthma. Our objective is to investigate the effectiveness of heat-sensitive moxibustion compared with conventional drug treatment.

**Methods/Design:**

This study is comprised of a multi-centre (12 centers in China), randomized, controlled trial with two parallel arms (A: heat-sensitive moxibustion; B: conventional drug). Group A selects heat- sensitive acupoints from the rectangle region which consist of two outer lateral lines of dorsal Bladder Meridian of Foot-Taiyang, and two horizontal lines of BL13(Fei Shu) and BL17 (Ge Shu);6 inch outer the first and second rib gap of anterior chest. Group B treats with fluticasone/salmeterol (seretide). The outcome measures will be assessed over a 3-month period before each clinic visit at days 15, 30, 60, and 90. Follow-up visit will be at 3, 6 months after the last treatment session. Adverse event information will be collected at each clinic visit.

**Discussion:**

This trial will utilize high quality trial methodologies in accordance with CONSORT guidelines. It may provide evidence for the effectiveness of heat-sensitive moxibustion as a treatment for chronic moderate persistent asthma. Moreover, the result may propose a new type moxibustion to control asthma.

**Trial Registration:**

The trial is registered at Chinese Clinical Trials Registry: ChiCTR-TRC-09000599

## Background

Asthma is a common chronic inflammatory disease of the airways characterized by variable and recurring symptoms, airflow obstruction, and bronchospasm [1]. It is also a complex disease involving many cells and mediators[2]. Asthma affects 300 million people worldwide[3], with an increasing prevalence in Western Europe(5%) and the USA(7%) in particular[4,5]. Despite the fact that there is still no cure for asthma, it has been established in a great number of small and large studies that many patients can reach a good asthma control with controller treatment [6]. Generally speaking, Medications used to treat asthma are divided into two general classes: quick-relief medications used to treat acute symptoms and long-term control medications used to prevent further exacerbation [7]. The therapeutic options available for patients with asthma depend on the severity of the condition. Although the symptoms can be controlled by drug treatment in most patients, effective low-risk, non-drug strategies could constitute a significant advance in asthma management [1-3]. Therefore, an increasing number of patients with asthma are attracted by complementary and alternative medicine (CAM) [8]. A survey showed that roughly 50% of asthma patients used some form of unconventional therapy [9].

Acupuncture has traditionally been used in asthma treatment in China and is increasingly applied for this purpose in Western countries. Moxibustion is a traditional Chinese method of acupuncture treatment, which utilizes the heat generated by burning Moxa (it is also called Mugwort or Moxa) to stimulate the acupuncture points. The technique consists of lighting a moxa-stick and bringing it close to the skin until it produces hyperemia due to local vasodilatation. The intensity of moxibustion is just below the individual tolerability threshold. Moxibustion has anti-inflammatory or immunomodulatory effects against chronic inflammatory conditions in humans [10]. Moxibustion method for treatment of asthma diverse curative effect and its mechanism may be due to improve lung function, antagonize of inflammatory mediators, modulate of immune function, regulate the role of cyclic nucleotide levels, and can also affect the neuroendocrine network and inflammatory cells[11-14]. Therefore, these inflammatory substances may be reduced and weakened by moxibustion. Especially for chronic persistent asthma, moxibustion may get a better effect.

Although firm evidence has not been established, the results of some clinical trials suggest that acupuncture and moxibustion may effective in the treatment of asthma [15-18]. However, these researches do not confirm the efficacy of acupuncture and moxibustion. This may be because all relevant RCTs were limited by methodological defects, including inappropriate sample size, variability of acupuncture and sham protocols, and missing information. Therefore, rigorous high-quality randomized controlled trials are needed.

Thinking about moxibustion itself, the selection of location for manipulating Moxa plays an important role in obtaining good effects [19]. The moxibustion point location may be connected with the changes in the condition of the disease. Our team of experts were astonished to find that the main factor in selecting the location of acupuncture points is link with the area which is affected by disease, not only the standardized fixed position.

In the human nature, there are two state of being in acupuncture points, the sensitized or awake state and the rest state. When the human body suffers form disease, the acupoints on the surface of the body are stimulated and sensitive by various stimulants including heat. The specific areas stimulated by heat are then called "heat-sensitive points". One of the characteristics of these areas is that they are specific or closely related to acupuncture points and have the same clinical effect as "a small stimulation induces a large response". The *Inner Canon of Huangdi *or *Yellow Emperor's Inner Canon *is an ancient Chinese medical text that has been treated as the fundamental doctrinal source for Chinese medicine for more than two millennia and until today. According with its core viewpoint and theory, acupoint is described and understood with the state, which is certain area of the body surface in the course of diseases. Among the changes, sensitized status is the common one, described that acupoints on the body surface may be sensitized in the course of diseases. Acupoint heat-sensitization is a type of acupoint sensitization. Our research found that the heat-sensitive phenomenon to a point or an area is a new type of reaction featured in a pathological state [20-23]. We applicated the acupoint heat-sensitization phenomenon and rule in the past twenty years. Our team experimental evidence indicates that the state of a point might change from the rest state to the heat sensitized state while suffering from diseases. Its characteristic was thought that these special acupoints might produce heat response and farther warm sensation, as a result of stimulation of moxibustion heat. If we can search out these heat-sensitized acupoints associating with pathological state, good effect will be achieved. Therefore, selecting the heat-sensitized acupoint may obtain therapeutic effect far better than acupuncture and moxibustion at acupoints of routine rest state. So we defined the approach which treated various diseases through heat-sensitized acupoint, as heat- sensitive moxibustion therapy. We carried out many clinical trials to test and verify the efficacy of heat-sensitized acupoint, such as myofascial pain syndrome [22], lumbar disc herniation [24], pressure sores [25] and knee osteoarthritis [26]. The result of clinical trials almost suggested superiority effect of heat-sensitized acupoint and encouraged us to proceed. Hence, we planned a rigorous multi-centre randomized controlled trial with a large sample size.

## Method/design

### Objective

The aim of this study is to investigate the effectiveness of heat-sensitive moxibustion compared with fluticasone/salmeterol (seretide) in patients with chronic moderate persistent asthma in China.

### Outcome measures

#### Primary outcome

At present, the goal of asthma care is to achieve and maintain control of clinical manifestation of the disease [27,28]. Hence, we use Asthma Control Test (ACT) to quickly access asthma control, a simple 5-question tool that is completed by the patient and parents/caregivers and recognized by the National Institutes of Health (Table 1)[29,30]. Patients should write the number of each answer in the score box provided, and then add up each score box for your total. ACT will be assessed before treatment and at days 15, 30, 60, and 90 in the treatment period. Follow-up visit will be at 3, 6 months after the last treatment session.

**Table 1:** Asthma Control Test (ACT) for people 12 yrs and older.

1. In the past 4 weeks, how much of the time did your asthma keep you from getting as much done at work, school or at home?					Score
All of the time ①	Most of the time ②	Some of the time ③	A little of the time ④	None of the time ⑤	

**2. During the past 4 weeks, how often have you had shortness of breath?**					

More than once a day ①	Once a day ②	3 to 6 times a week ③	Once or twice a week ④	Not at all ⑤	

**3. During the past 4 weeks, how often did your asthma symptoms (wheezing, coughing, shortness of breath, chest tightness or pain) wake you up at night or earlier than usual in the morning?**					

4 or more nights a week ①	2 or 3 nights a week ②	Once a week ③	Once or twice ④	Not at all ⑤	

**4. During the past 4 weeks, how often have you used your rescue inhaler or nebulizer medication (such as albuterol)?**					

3 or more times per day ①	1 or 2 times per day ②	2 or 3 times per week ③	Once a week or less ④	Not at all ⑤	

**5. How would you rate your asthma control during the past 4 weeks?**					

Not controlled at all ①	Poorly controlled ②	Somewhat controlled ③	Well controlled ④	Completely controlled ⑤	

#### Secondary outcomes

Measurement of lung function provides an assessment of the severity, reversibility, and variability of airflow limitation. Forced expiratory volume in 1 s (FEV1) and peak expiratory flow (PEV) will be used in this trial. Attack frequency also will be assessed. These outcomes also will be assessed before treatment and at days 90 in the treatment period. Follow-up visit will be at 3, 6 months after the last treatment session. Adverse event information will be collected at each clinic visit.

### Design

A multi-centre, randomized, two parallel arms (group A and B) and assessor blinded, positive controlled trial will be conducted at the twelve centers in China (Table 2).

**Table 2:** List of 12 clinical centers

Name	City	Province
Affiliated Hospital of Jiangxi University of Traditional Chinese Medicine(TCM)	Nanchang	Jiangxi

Guangdong Hospital of Traditional Chinese and Western Medicine	Foshan	Guangdong

Wuhan the First Hospital	Wuhan	Hubei

Guangdong Hospital of TCM	Guangzhou	Guangdong

Guangzhou University of TCM	Guangzhou	Guangdong

Guangdong Disabled Soldier Hospital	Guangzhou	Guangdong

Affiliated Hospital of Shandong University of TCM	Jinan	Shandong

Suzhou Hospital of TCM	Suzhou	Jiangsu

The First Affiliated Hospital of Zhejiang University of TCM	Hangzhou	Zhejiang

The First Affiliated Hospital of Chongqing Medical University	Chongqing	Chongqing

The First Affiliated Hospital of Nanchang University	Nanchang	Jiangxi

Nanchang Hospital of Traditional Chinese and Western Medicine	Nanchang	Jiangxi

**Figure 1:**
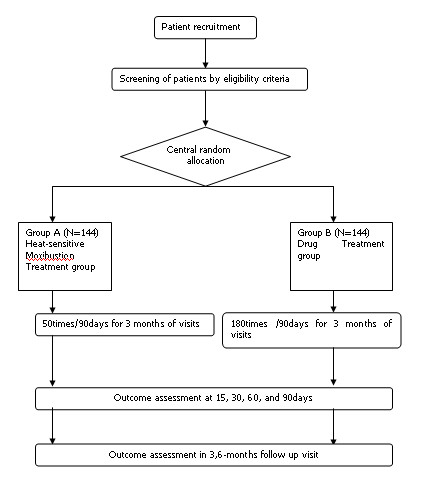
**The flow diagram is intended to depict the passage of participants through this RCT**.

The study will be sequentially conducted as follows: a run-in period of one week prior to randomization, a treatment period of 90 days, and a follow-up period of six months. At the end of the run-in period, participants will be randomized to the heat-sensitive moxibustion group or the drug group by the central randomization system (Figure 1). This system is provided by China Academy of Chinese Medical Sciences, which adopted the computer telephone integration (CTI) technology to integrate computer, internet and telecom. The random number list will be assigned by interactive voice response (IVR) and interactive web response (IWR) [31]. The success of blinding will be assessed at each participant's last visit. Assessor who did not participate in the treatment and who is blinded to the allocation results will perform the outcome assessment.

### Eligibility

#### Inclusion criteria

According to the guideline of asthma treatment and prevention in China (GATPC) [32], patients are divided into three types, including acute exacerbation, chronic persistent and clinical remission. Severity grade of asthma in GATPC is described in Table 3. Our trial will only choose participants with chronic moderate persistent asthma (Figure 1).

**Table 3:** Clinical classification of severity in GATPC

Characteristic	Intermittent (Grade I)	Mild (Grade II)	Moderate (Grade III)	Severe (Grade IV)
**symptoms**	Less than once week	More than once week, < once a day	Everyday	Everyday

**Limitation of activities and sleep**	Transient	May be affect activities and sleep	Affect activities and sleep	Frequent

**Nocturnal of symptoms/awakening**	Less than twice a month	More than twice a month, < once a week	More than once week	Frequent

**Lung function(PEF or FEV1)**	≥80% predicted (FEV1)or personal best (PEF); mutation rate < 20%	≥80% predicted (FEV1)or personal best (PEF); mutation rate 20%~30%	60%~79% predicted (FEV1)or personal best (PEF); mutation rate > 30%	< 60% predicted (FEV1)or personal best (PEF); mutation rate > 30%

Patients will be required to complete the baseline asthma diary. Written informed consent will be obtained from each participant. Participants 18~65 years of age will be recruited from outpatient and inpatient in 12 centers. Standard of diagnosis listed as follows: (1) Common signs and symptoms of asthma include: recurrent wheezing, coughing, trouble breathing, chest tightness; (2) Symptoms that occur or worsen at night, and symptoms that are triggered by cold air, exercise or exposure to allergens; (3) Scattered or diffuse expiratory wheezing sound could be heard in the lungs in attack; (4) The above symptoms will be relieved or disappear after treatment; (5) Ruling out conditions other than asthma; (6) When typical clinical symptoms can not be observed, lung function tests should be used to confirm, such as positive challenge test, positive bronchodilator test(an increase in FEV1 of ≥12% and ≥200 ml), and mutation rate of PEF≥20% one day/two weeks. Since the research involves moderate asthma (grade III), the inclusion criteria will restrict the following conditions: According to the below GATPC diagnosis standard, meanwhile, heat-sensitivity appears within the rectangle area which consist of two outer lateral lines of dorsal Bladder Meridian of Foot-Taiyang, and two horizontal lines of BL13 (Fei Shu) and BL17 (Ge Shu); 6 inch outer the first and second rib gap of anterior chest. Participants will be instructed to stop asthma symptomatic relief medication during the run-in and treatment periods and will be provided the usual care instruction for asthma.

#### Exclusion criteria

Participants will be excluded if they have other diseases that also cause breathlessness or dyspnea, such as bronchiectasis, cor-pulmonale, pulmonary fibrosis, tuberculosis, pulmonary abscess, chronic obstructive pulmonary disease and so on. Participants will not be eligible if the female are in the duration of pregnancy or lactation. The following conditions are also excluded items: Complicated with serious life-threatening diseases, such as heart and brain blood vessels, liver, kidney and hematopoietic system disease and psychotic patients; Hormone-dependent type patients, or the people who used adrenal cortical hormone (intravenous, intramuscular injection, subcutaneous injection and oral administration) within 4 weeks before recruiting.

### Treatment protocol

#### Heat-sensitive moxibustion

Moxibustion will be performed by certified acupuncture medical doctors at 12 centers. Qualified specialists of acupuncture in traditional Chinese medicine with at least five years of clinical experience will perform the acupuncture in this study. All treatment regimens will be standardized between 12 centers practitioners via video, hands-on training and internet workshops. Participants will be randomly assigned to the heat-sensitive moxibustion group, or the drug group. In the former group, a 22 mm (diameter) × 160 mm (length) moxa-sticks (Jiangxi Hospital of Traditional Chinese Medicine, China) will be used. The patient is usually in the comfortable position for treatment, with 24°C~30°C temperature in the room. He should wear loose clothes.

For the heat-sensitive moxibustion group, the moxa-sticks are lit by the therapist and held over the rectangle area which consist of two outer lateral lines of dorsal Bladder Meridian of Foot-Taiyang, and two horizontal lines of BL13(Fei Shu) and BL17 (Ge Shu);6 inch outer the first and second rib gap of anterior chest. The warming suspended moxibustion about distance of 3 cm above the skin are used to search the acupoint heat-sensitization phenomenon. The following patients sensation will suggest the special heat-sensitization acupoint: diathermanous sensation due to moxa-heat, defining as the heat sensation conducting from the Moxa local skin surface into deep tissue, or even into the thoracic cavity; expand heat sensation due to moxa-heat, defining as the heat sensation spreading the surrounding little by little around the moxa point; transfer heat sensation due to moxa-heat, defining as the heat sensation transferring along some pathway, or even to the arms. The therapists mark the point as heat-sensitive acupoint. We try our best to seek all the special acupoints in each patient by the repeated manipulation.

The therapists begin to treat patients from the most heat-sensitive intensity acupoint. Treatment sessions end when patients are feel the acupoint heat-sensitization phenomenon disappeared. Generally speaking, we find the time range from 30~60 minutes. In 1^st ^month, patients receive the treatment once a day in the first eight days, and 12 times treatment in the next twenty-two days. Treatments will be given 15 times a month for the remaining two months.

#### Drug group

In recent years, considerable insight has been gained in to the optimal management of adult asthma. Those with persistent asthma are usually not well controlled without inhaled corticosteroids (ICS). Adding a long-acting beta-agonist (LABA) to ICS appears to be well controlled [33]. The combination of an ICS and LABA is preferred in these patients, and is better than doubling or even quadrupling the dose of ICS to achieve better asthma control and reduce exacerbation risks [34,35]. Two such combinations, salmeterol xinafoate and fluticasone propionate (SFC, Seretide(tm)) and formoterol and budesonide (FBC, Symbicort(tm)) are widely used and have been shown to be effective in controlling asthma of varying severity in adults and children[36-38].

Therefore, we selected the Seretide Accuhaler, containing two medicines, fluticasone propionate and salmeterol xinafoate, which is the mainstay of current asthma treatment. This drug is recommended by GINA as a regular treatment. Our trial uses salmeterol/ﬂuticasone 50 μg/250 μg twice a day. Patients receive the treatment for a total of 180 sessions over 90 days.

### Statistical methods

#### Statistical analysis plan

We will conduct analysis on an intention-to-treat basis, including all randomized participants with at least one measurable outcome report. Analyses will be conducted using 2-sided significance tests at the 5% significance level. An analysis using the Cochran-Mantel-Haenszel procedure will be done to asses center effect. The statistician conducting the analyses will remain blind to treatment group and data will only be unblinded once all data summaries and analyses are completed. All analyses will be conducted in the SAS statistical package program (ver. 9.1.3).

#### Baseline data

Baseline characteristics will be shown as mean ± standard deviation (SD) for continuous data including age, previous duration, and so on. As for participants' gender, n (%) of male and female in each group will be shown as baseline characteristics. We will conduct between-group comparison in baseline using two-sample t-test or Wilcoxon rank sum test for continuous data and using Chi-square test or Fisher's exact test for gender composition considering p < 0.05 as statistically significant.

If any imbalances in baseline characteristics between groups are encountered, we will conduct ANCOVA (analysis of covariance) using these imbalanced variables as covariates and allocated group as fixed factor.

#### Outcome data

For primary and secondary outcome measures, these will be summarized descriptively (mean, SD, median, minimum and maximum) at each time point by treatment group. The t-test, Mann-Whitney U and Wilcoxon test were used for comparison of variables, as appropriate.

All adverse events reported during the study will be included in the case report forms; the incidence of adverse events will be calculated. The percentage of subjects with adverse events in each group will be calculated and compared using the chi-squared test or Fisher's exact test.

##### Dropped or missing data

Reasons for dropped or missing data will be explored by descriptively. Missing data will be replaced according to the principle of the last observation carried forward.

##### Follow-up data

The primary outcome will be the ACT scores. The primary analysis will compare the two groups in 3 months. A secondary analysis will compare the two groups at 6,9 months to assess if any differences between groups have been maintained over time.

Loss to follow-up is likely to lead to biased estimates of intervention effect. We will try to avoid bias due to attrition by carefully following up the participants in both groups. We will phone participants who fail to complete questionnaires after a second reminder. We anticipate a 20% loss to follow-up in this trial, and will implement procedures to minimize loss to follow-up and patient withdrawal, and where possible, we will collect information on reasons for patient withdrawal.

#### Data integrity

The integrity of trial data will be monitored by regularly scrutinizing data sheets for omissions and errors. Data will be double entered and the source of any inconsistencies will be explored and resolved.

#### Sample size

We wished to estimate the sample size according to non-inferiority clinical trial between the heat-sensitive and drug group. Sample size depends on the level of confidence chosen, the risk of type II error (or desired power), and δ. The parameter margin of non-inferiority δ can be specified as a difference in means or proportions. It is often chosen as the smallest value that would be a clinically important effect. To determine δ, we carried out a small sample pilot study previously. The primary endpoint chosen was ACT. The result of outcome showed difference in means between the two groups approximately was 0.45. The choice of δ = 0.15 (30% of Δ) appeared to be reasonable based on clinical relevance and statistical judgment.

If we apply a two-sided 5% significance level(δ = 0.15, α = 0.05, β = 0.2), 95% power the calculated required sample size is approximately 120 participants in each group, according to the following equation. Allowing for a 20% loss to follow up, a total of 144 participants will be required in each group, with 288 participants in total.

$$n=2\times \frac{{\left(U_{\alpha }+U_{\beta }\right)}^{2}}{\gamma^{2}}\times P(1-P)$$

### Adverse events

We define adverse events as unfavorable or unintended signs, symptoms or disease occurring after treatment that are not necessarily related to the moxibustion intervention. In every visit, adverse events will be reported by participants and examined by the practitioner.

### Ethics

Written consent will be obtained from each participant. This study was approved by all relevant local ethics review boards. Ethics Committee of Affiliated hospital of Jiangxi Institute of Traditional Chinese Medicine had approved this trial: code issued by ethic committee is 2008(13).

## Discussion

To our knowledge, the goal of asthma care is to achieve and maintain control of clinical manifestations of the disease for prolong periods. When asthma is controlled, patients can prevent most attacks, avoid troublesome symptoms day and night, and keep physically active [1]. Acupuncture therapy was often perceived as an effective option to control asthma successfully by patients with chronic asthma. The use of acupuncture in asthma patients is increasing as an adjunct and also as a substitute for effective and proven therapies [39]. In China, moxibustion are considered as an ancient treatment to prevent and control asthma, and still widely used today. A number of clinical trial suggested moxibustion as one of traditional acupuncture therapy, should effective in the treatment of asthma. But the methodological problems of published trials haunt us the trust of moxibustion. Therefore, we design this rigorous clinical trials meeting the CONSORT statement and guidelines to guarantee a high internal validity for the results.

At present, various conventional medications are used to slow down and control with the disease. An ICS/LABA combination in a single inhaler represents a safe, effective and convenient treatment option, and recommended by GINA. So, we selected the fluticasone/salmeterol (seretide) as the control treatment in the protocol. Actually, the aim of this trial is to search an effective CAM treatment to control asthma, as good as conventional drug. The focused features of moxibustion are low cost, less adverse event and low risk.

According to the current theory of traditional Chinese medicine, moxibustion resulting from the burning of Moxa produces the radiant heat and drug effects to acupoints. This treatment penetrates deeply into the body, restoring the balance and flow of vital energy or life force through acupoints. So the selected of location for manipulating Moxa plays an important role in obtaining good effects. Generally speaking, the location acupoints are fixed along meridians. The conventional moxibustion is considered as improving general health and treating diseases by stimulating these fixed acupoints. And doctors consider hyperemia due to local skin vasodilatation as the indicator of moxibustion's effect. However, our clinical experience and observation in the past suggested that stimulating these fixed acupoints might not the best treatment site for moxibustion. In the human nature, there are two states of acupoints, the stimulated or awake state and the rest state. When the human body suffers form disease, the acupuncture points on the surface of the body are stimulated and sensitive by various stimulants including heat. And acupoint heat-sensitization is a type of acupoint sensitization. Acupoint is more than fixed skin site but external sensitive point reflecting the diseases. Therefore, acupoint is variable and depends on the pathological state. Traditional fixed acupints are thought as indicators to searching specific sensitive acupoint. That is, traditional fixed acupints don not consider the state as the key factor to local the acupoint, so the course of fixing the position is imprecisely.

When we light the Moxa hold over the heat-sensitive acupoints, the patients will produce some heat-sensitization phenomenon. The following patients sensation will suggest the special heat-sensitization acupoint: diathermanous sensation due to moxa-heat, defining as the heat sensation conducting from the moxa local skin surface into deep tissue, or even into the thoracic cavity; expand heat sensation due to moxa-heat, defining as the heat sensation spreading the surrounding little by little around the moxa point; transfer heat sensation due to moxa-heat, defining as the heat sensation transferring along some pathway, or even to the arms.

Acupuncture and moxibustion originated in China several thousands of years ago. The ancient Chinese medical classic *Huáng Dì Nèi Jīng *translated as *'The Yellow Emperor's Inner Classic' has *been treated as the fundamental doctrinal source for Chinese medicine for more than two thousand years. According to the chapter *Annotations on 'The Yellow Emperor's Inner Classic - chapter: jiu zhen shi er yuan'(translated as 'Nine Needles and twelve yuan-primary acupoints")*, it says: *"the so-called joints are the places where Shenqi flows in and out, not just referring to skin, muscles, sinews and bones." *This explains that the acupuncture points are not located according to the flesh and bones, which by definition have a fixed location, but they are alive and have a dynamic state due to the activity of "shen-qi". In the Huáng Dì Nèi Jīng Ling Shu (黄帝内经灵枢) translated as "*Annotations on 'The Yellow Emperor's Inner Classic' - chapter: jiu zhen shi er yuan(translated as 'Nine Needles and twelve yuan-primary acupoints")*, it says: "so the disease of the Five Zang-organs can be treated by needling the twelve Yuan-Primary acupoints. The twelve Yuan-Primary acupoints show how the five Zang-Organs receive the nutrients of food and water and how Essence-Qi is infused into the three hundred and sixty-five joints. That is why the disease of the Five Zang-Organs are manifested over the twelve Yuan-Primary acupoints which show certain manifestations. Awareness of the twelve Yuan-Primary and observation of the manifestation to know the pathological changes of the Five Zang-Organs". We can learn an important fact from this section of the classical text. It clearly states that acupuncture points reflect the pathological state of the internal diseases and can be stimulated in treatment. Physically, people are not always aware of the existence of the acupuncture points. In contrast, patients can usually feel some changes in the area of the acupuncture point when affected by disease. Through the observation of these changes, the ancient doctors located the acupuncture points. In the *Annotations on 'The Yellow Emperor's Inner Classic - chapter: 'back-shu *acupoints', it states: "The Feishu (BL13) acupoint is located between the third thoracic vertebra. The Xinshu (BL15) acupoint is located below and lateral to the fifth thoracic vertebra. The Geshu (BL17) acupoint is located below and lateral to the seventh thoracic vertebra. The Ganshu (BL18) acupoint is located below and lateral to the ninth thoracic vertebra. The Pishu (BL20) acupoint is located below and lateral to the eleventh thoracic vertebra. The Shenshu (BL23) is located below and lateral to the fourteenth thoracic vertebra. These acupoints are all located beside the spinal column and 3 cun away from the spinal column. The method to locate these acupoints is to press the regions. When pressed, the patient will feel aching and distending or feel that the original pain is relieved." This section of the classical text illustrated that the back shu points are found by founding the sensitive areas on the skin. In the *Annotations on 'The Yellow Emperor's Inner Classic - chapter: 'five xie' *(translated as 'five kinds of pathogenic factor')it says: *"cough involving the shoulder and back, to treat such a disease, acupoints located on the lateral side of the chest and lateral to the third thoracic vertebra can be needled. Before applying acupuncture, the doctor may use his fingers to quickly press the concerned region; the place where the patient feels comfortable when pressed is the acupoint and should be needled"*. From this section, we can conclude that the sensitivity of the points is the key factor to locate the position of the acupuncture points.

Among the changes, sensitized status is the common one, described that acupoints on the body surface may be sensitized with various types of sensitization. This sensitized acupoint is not only the pathological phenomenon reflecting the diseases but also stimulating location with acupuncture and moxibustion. Acupoint heat-sensitization is a type of acupoint sensitization, which derived from our clinical experience and research in past twenty years. The special acupoint makes accordance with the classical thought and theory from the Inner Canon of Huangdi.

Our empirical evidence engaged us to formulate the following hypothesis: selecting the heat-sensitized acupoint may obtain therapeutic effect in asthma. The main aim of this trial is to test and verify the hypothesis. If we can confirm this hypothesis, the results of our trial will be helpful to supply the evidence on searching better safe approach to control asthma.

## Competing interests

The authors declare that they have no competing interests.

## Authors' contributions

RC and MC obtained funding for the research project and drafted the protocol. JX wrote the final manuscript. RC contributed to the research design and made critical revisions. ZC and BZ were responsible for the statistical design of the trial and wrote portions of the statistical methods, data handling, and monitoring sections. All authors read and approved the final manuscript.
